# Silent Struggles: Sexual Dysfunction Among Breast Cancer Survivors From Three Tertiary Hospitals in Tunisia

**DOI:** 10.7759/cureus.94774

**Published:** 2025-10-17

**Authors:** Mouna Kouira, Ekram Guerbej, Riadh Ncibi, Ines Mkhinini, Mohamed Raouf Ben Abdesslem, Hafedh Abbassi, Latifa Lassoued

**Affiliations:** 1 Department of Obstetrics and Gynecology, Farhat Hached University Hospital/Faculty of Medicine Ibn El Jazzar of Sousse, Sousse, TUN; 2 Department of Obstetrics and Gynecology, Ibn El Jazzar University Hospital/Faculty of Medicine Ibn El Jazzar of Sousse, Kairouan, TUN; 3 Department of Obstetrics and Gynecology, Sidi Bouzid University Hospital/Faculty of Medicine Ibn El Jazzar of Sousse, Sidi Bouzid, TUN

**Keywords:** breast cancer, fsfi, quality of life, sexual dysfunction, survivorship, tunisia

## Abstract

Background

Breast cancer is the most common malignancy among women worldwide and the leading cause of cancer-related mortality. While survival rates have improved, the impact of breast cancer and its treatments on female sexual health remains underexplored, particularly in North African populations.

Objective

The objective of this study is to assess the prevalence and determinants of sexual dysfunction among women in remission after treatment for non-metastatic breast cancer in Tunisia.

Methods

We conducted a descriptive, cross-sectional multicenter study (November-December 2024) including 200 sexually active women in remission from non-metastatic breast cancer recruited from three tertiary hospitals in Tunisia. Sexual function was assessed using the validated Arabic version of the Female Sexual Function Index (FSFI-19). Sociodemographic, clinical, and treatment variables were collected and analyzed with standard statistical tests; sexual dysfunction was defined as a total FSFI score ≤26.55.

Results

The mean age was 50.3 ± 23.2 years; 74% were aged 41-67 years. Most women (83%) underwent surgery, 92% chemotherapy, 80% radiotherapy, and 55% hormonotherapy; only 10% had breast reconstruction. The mean FSFI score was 18.3 ± 11.6, with 87% of participants meeting criteria for sexual dysfunction. Desire (59%) and arousal (59.5%) were the most impaired domains, while 43% reported lubrication difficulties, 50% had orgasmic dysfunction, and 56.5% had dyspareunia. Overall, 74.5% of participants reported sexual dissatisfaction. Analytically, sexual dysfunction was more frequent after radical surgery compared to conservative surgery (91% vs. 78%, p=0.04), and among women receiving radiotherapy (92% vs. 76%, p=0.02) or hormonotherapy (94% vs. 78%, p=0.01). Radiotherapy was associated with more lubrication difficulties and sexual pain, while hormonotherapy mainly impaired desire and lubrication. No significant differences were observed for chemotherapy.

Conclusion

Sexual dysfunction is highly prevalent among Tunisian breast cancer survivors, particularly affecting desire and arousal. Specific treatments, especially radical surgery, radiotherapy, and hormonotherapy, exacerbate sexual impairments. Systematic assessment of sexual health using validated instruments such as the FSFI should be integrated into routine oncology follow-up. Multidisciplinary, culturally adapted interventions are urgently needed to address the physical, psychological, and relational dimensions of survivorship care.

## Introduction

Cancer remains the second leading cause of death worldwide, accounting for an estimated 9.7 million deaths in 2023, or nearly one in eight deaths [1]. Among all cancer types, breast cancer is the most common globally and continues to be the leading cause of cancer-related mortality among women. According to the World Health Organization (WHO), approximately 2.3 million women are diagnosed with breast cancer each year, representing 11.6% of all cancer cases [2].

In Tunisia, breast cancer is the most frequent malignancy in women and the primary cause of female mortality between 35 and 55 years of age [3]. Its high incidence, severity, and the profound physical and psychological sequelae associated with the disease make it a major public health concern [4].

Sexuality is a particularly vulnerable domain in breast cancer survivors. Beyond the biological impact of treatments, women frequently face fear of infertility, negative body image, loss of femininity, diminished sexual attractiveness, depression, anxiety, and altered sexual self-concept [5]. Despite its importance, sexuality remains one of the most underexplored and underaddressed dimensions of survivorship care. Patients seldom raise sexual concerns directly, and healthcare providers rarely initiate discussions on this topic [4,5].

Given this gap, we conducted a multicenter study to evaluate the sexual health of women in remission following treatment for non-metastatic breast cancer.

## Materials and methods

We conducted a descriptive, cross-sectional, multicenter study over a two-month period (November-December 2024). Data collection was performed in three tertiary care centers in Tunisia: the Gynecology Department of Ibn El Jazzar University Hospital (Kairouan), the Oncology and Gynecology Departments of the Military Hospital of Tunis, and the Gynecology Department of Fattouma Bourguiba University Hospital (Monastir). Eligible participants were women with a history of histologically confirmed, non-metastatic breast cancer who were in complete remission and attending routine outpatient follow-up visits.

Inclusion criteria were age ≤70 years, the completion of adjuvant chemotherapy and/or radiotherapy at least three months before enrollment, sexual activity defined as partnered sexual intercourse within the preceding four weeks, and the ability to provide informed consent and respond to the questionnaire in Arabic. Exclusion criteria were the presence of metastatic disease or cancer recurrence, a severe psychiatric comorbidity or cognitive impairment interfering with survey completion, and refusal or inability to participate.

A non-probability convenience sampling strategy was applied. During the study period (November-December 2024), consecutive eligible women were invited to participate. A total of 200 patients were enrolled: 50 from Ibn El Jazzar University Hospital, 100 from the Military Hospital of Tunis (40 from oncology, 60 from gynecology), and 50 from Fattouma Bourguiba University Hospital. All participants provided written informed consent.

A priori sample-size considerations were based on the standard formula for estimating a prevalence in cross-sectional studies: $n = \frac{Z^{2} p (1-p)}{d^{2}}$, where Z is the standard normal deviate at 95% confidence interval (CI) (1.96), p is the expected prevalence, and d is the desired precision. Based on a Tunisian study reporting a prevalence of sexual dysfunction among breast cancer survivors of approximately 70-73% [6], the required sample size would be about 301 for a precision of ±5% and 154 for a precision of ±7%. The achieved sample (n = 200) therefore provided an acceptable precision level for descriptive and univariate analyses, despite the non-probability nature of recruitment. 

Data were collected using a structured, anonymous questionnaire administered in Arabic. The instrument captured: (i) Sociodemographic variables: age, marital status, education level, occupation, partner’s education, parity, and menopausal status and (ii) Clinical and therapeutic variables: tumor size and stage, lymph node status, surgical procedures (classified as breast-conserving surgery versus radical mastectomy, with or without axillary dissection), reconstruction, chemotherapy, radiotherapy, hormonotherapy, and treatment tolerance.

Sexual function was evaluated using the validated Arabic version of the Female Sexual Function Index (FSFI-19), a 19-item self-administered questionnaire widely used in sexual medicine. The Arabic-Lebanese version (FSFI-LB) demonstrated excellent psychometric properties, with strong internal consistency (Cronbach’s α = 0.973) and excellent test-retest reliability (r = 0.9997), confirming its validity for use in Arabic-speaking populations [7]. The FSFI explores six domains of female sexual function over the preceding four weeks: desire (range 1.2-6), arousal (0-6), lubrication (0-6), orgasm (0-6), satisfaction (0.8-6), and pain (0-6). Scores from each domain are summed to generate a total score ranging from 2 to 36, with higher scores indicating better sexual function. A total FSFI score ≤26.55 was used to define female sexual dysfunction (FSD). For domain-specific analyses, dysfunction was defined as a score below 33% of the maximum possible score in each domain.

A pilot pretest was performed on 20 women at Ibn El Jazzar University Hospital to ensure clarity and cultural appropriateness of the questionnaire. These data were excluded from the final analysis.

Data were entered and analyzed using IBM SPSS Statistics for Windows, Version 21 (Released 2012; IBM Corp., Armonk, New York, United States). Continuous variables were summarized as means ± standard deviation (SD) or medians with interquartile ranges (IQR) depending on distribution. Categorical variables were expressed as frequencies and percentages. The prevalence of sexual dysfunction was estimated with 95% CIs. Univariate analysis was conducted to estimate odds ratios (OR) with 95% confidence intervals for global sexual dysfunction (FSFI ≤26.55). Associations between FSD and sociodemographic or clinical variables were explored using chi-square or Fisher’s exact tests for categorical data and t-test or Mann-Whitney U test for continuous data. A p-value <0.05 was considered statistically significant.

The study protocol was approved by the institutional ethics committees of the participating hospitals. All procedures complied with the Declaration of Helsinki. Written informed consent was obtained from each participant prior to enrollment, and anonymity and confidentiality were strictly maintained.

## Results

Socio-demographic characteristics:

Most patients (74%) were between 41 and 67 years old, while 26% were between 20 and 40 years old. The mean age was 50.35 ±23.22 years. Socio-demographic characteristics are summarized in Table 1.

**Table 1:** Participants' characteristics

Characteristics	N (%)
Age	
20-40	52 (26%)
41-67	148 (74%)
Geographic origins	
Urban areas	120 (60%)
Rural areas	80 (40%)
Education level	
College	80 (40%)
High school	30 (15%)
Primary school	40 (25%)
Illiterate	50 (25%)
Partner's educational level	
College	88 (44%)
High school	12 (6%)
Primary school	53 (26.5%)
Illiterate	47 (23.5%)
Professional activities	
Active	66 (33%)
On sick leave.	64 (32%)
Unemployed	70 (35%)
History of infertility	62 (31%)
Primary infertility	4 (%)
≥ 1 living child	90.62%
Postmenopausal status	140 (70%)
Natural	76 (54%)
Induced	64 (46%)

Tumor characteristics and treatment

On the TNM staging system, 61.5% of patients had T2 tumors. Seventy percent had no lymph node involvement (N0), and only a minority had advanced nodal stages (N1 to N3).

The vast majority of patients (83%) underwent surgical treatment. Among these surgeries, nearly three-quarters (73%) were radical procedures, while 27% were conservative. Breast reconstruction remained uncommon, performed in only 20 patients (10%).

Chemotherapy and radiotherapy were administered in 92% and 80% of cases, respectively. Fifty-five percent of patients received hormonotherapy.

Study of sexuality

No patient had refused to reply to the questionnaire. The mean FSFI score was 18.34 ±11.62 (ranging from 2 to 36). Overall, sexual dysfunction was 87% in our population.

By domain, the mean scores ranged from 2.8 to 3.34. The lowest scores were observed for desire (2.9±1.97) and arousal (2.8±1.97±1.97), while the highest score was noted for pain (3.34±2.16). 

**Figure 1:**
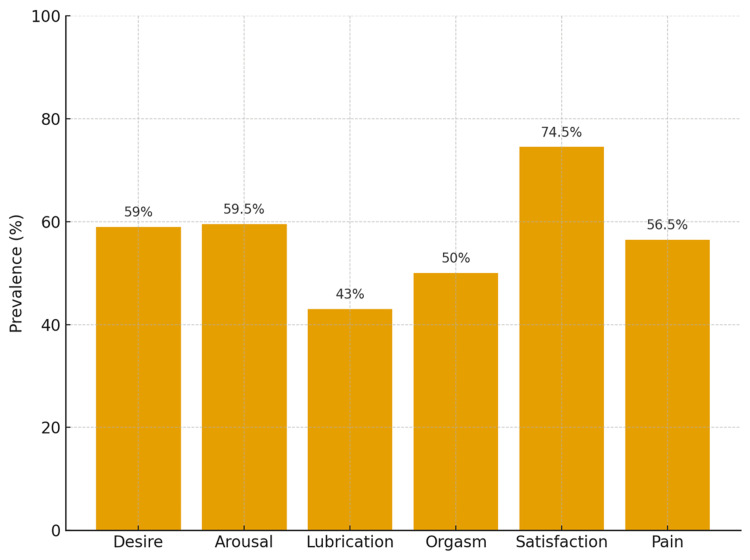
Prevalence of sexual dysfunction by the FSFI domain This figure indicates that sexual function was affected in several domains in more than 50% of the participants. FSFI: Female Sexual Function Index

Fifty-nine percent of patients had dysfunction in the desire domain, and 41% indicated very low or absent desire. Sexual pain was reported as bothersome by 56.5% of patients. Furthermore, 74.5% were dissatisfied with their sexual life, and 59.5% considered their arousal level to be average or low. Problems with lubrication were reported by 43% of women, while achieving orgasm was problematic for 50% of respondents (Figure 1).

Analytical study of the effect of different treatments on sexuality

A total of 200 women were included in the study. Among the 166 patients who underwent surgery, 121 (73%) had radical procedures and 45 (27%) conservative procedures.

Global sexual dysfunction was reported in 91% of radical cases compared with 78% of conservative cases (p = 0.04). Desire and arousal mean scores were lower in the radical group (2.7 ± 1.9 vs. 3.4 ± 1.9 and 2.6 ± 1.9 vs. 3.3 ± 2.0, respectively; p < 0.05). Differences in orgasm (54% vs. 39%), lubrication (45% vs. 39%), and pain (3.2 ± 2.1 vs. 3.5 ± 2.2) did not reach statistical significance (p > 0.05). Univariate analysis showed that radical (non-conserving) surgery was associated with a higher likelihood of global sexual dysfunction (FSFI ≤ 26.55) compared with conservative surgery (OR 2.86, 95% CI 1.12-7.29, p = 0.03) (Table 2).

**Table 2:** Sexual dysfunction according to surgery type (n=166) SD: Standard Deviation; OR: Odds Ratio; CI: Confidence Interval; FSFI: Female Sexual Function Index OR (95% CI) derived from univariate logistic regression for global sexual dysfunction (FSFI ≤ 26.55). * p < 0.05 is considered statistically significant.

FSFI Domain	Radical surgery (n=121)	Conservative surgery (n=45)	p-value	OR (95 % CI)
Global dysfunction	91%	78%	0.04*	2.86 (1.12–7.29)
Desire (mean ± SD)	2.7 ± 1.9	3.4 ± 1.9	0.02*	—
Arousal (mean ± SD)	2.6 ± 1.9	3.3 ± 2.0	0.03*	—
Orgasm dysfunction	54%	39%	0.10	—
Lubrication issues	45%	39%	0.22	—
Pain (mean ± SD)	3.2 ± 2.1	3.5 ± 2.2	0.21	—

Among 160 patients who received radiotherapy and 40 who did not, global dysfunction was more common in irradiated women (92% vs. 76%, p = 0.02). Lubrication difficulties (48% vs. 31%, p = 0.03) and sexual pain (3.1 ± 2.1 vs. 3.7 ± 2.2, p = 0.04) were significantly more frequent in irradiated patients. The mean FSFI total score was lower in the radiotherapy group (17.2 ± 11.3 vs. 22.5 ± 10.9, p = 0.01). Desire (2.8 ± 1.9 vs. 3.5 ± 2.0) and arousal (2.7 ± 1.9 vs. 3.5 ± 2.0) were also reduced. Radiotherapy was associated with higher odds of global sexual dysfunction compared with no radiotherapy (OR 3.77, 95% CI 1.51-9.39, p = 0.02) (Table 3).

**Table 3:** Sexual dysfunction according to radiotherapy (n=200) SD: Standard Deviation; OR: Odds Ratio; CI: Confidence Interval; FSFI: Female Sexual Function Index OR (95% CI) derived from univariate logistic regression for global sexual dysfunction (FSFI ≤ 26.55). * p < 0.05 is considered statistically significant.

FSFI Domain	Radiotherapy (n=160)	No Radiotherapy (n=40)	p-value	OR (95 % CI)
Global dysfunction	92%	76%	0.02*	3.77 (1.51–9.39)
Desire (mean ± SD)	2.8 ± 1.9	3.5 ± 2.0	0.06	—
Arousal (mean ± SD)	2.7 ± 1.9	3.5 ± 2.0	0.07	—
Orgasm dysfunction	53%	41%	0.09	—
Lubrication issues	48%	31%	0.03*	—
Pain (mean ± SD)	3.1 ± 2.1	3.7 ± 2.2	0.04*	—

Chemotherapy was administered to 184 patients, while 16 did not receive it. Global dysfunction was present in 88% of chemotherapy cases compared to 82% of non-chemotherapy cases (p = 0.33). Desire (2.8 ± 1.9 vs. 3.6 ± 2.0) and arousal (2.7 ± 1.9 vs. 3.5 ± 2.0) scores were lower among chemotherapy patients, though differences were not statistically significant. Pain scores were similar (3.3 ± 2.1 vs. 3.5 ± 2.2, p > 0.05). Chemotherapy did not show a significant association with global sexual dysfunction (OR 1.70, 95% CI 0.45-6.44, p = 0.33) (Table 4).

**Table 4:** Sexual dysfunction according to chemotherapy (n=200) SD: Standard Deviation; OR: Odds Ratio; CI: Confidence Interval; FSFI: Female Sexual Function Index OR (95% CI) derived from univariate logistic regression for global sexual dysfunction (FSFI ≤ 26.55). * p < 0.05 is considered statistically significant.

FSFI Domain	Chemotherapy (n=184)	No Chemotherapy (n=16)	p-value	OR (95 % CI)
Global dysfunction	88%	82%	0.33	1.70 (0.45–6.44)
Desire (mean ± SD)	2.8 ± 1.9	3.6 ± 2.0	0.08	—
Arousal (mean ± SD)	2.7 ± 1.9	3.5 ± 2.0	0.09	—
Orgasm dysfunction	50%	41%	0.22	—
Lubrication issues	43%	37%	0.25	—
Pain (mean ± SD)	3.3 ± 2.1	3.5 ± 2.2	0.19	—

One hundred and ten patients received hormonotherapy, and 90 did not. Global dysfunction was significantly higher among hormonotherapy users (94% vs. 78%, p = 0.01). Desire (2.7 ± 1.9 vs. 3.3 ± 2.0, p = 0.03) and lubrication (51% vs. 33%, p = 0.04) were significantly impaired. Orgasm (56% vs. 41%) and arousal (2.6 ± 1.9 vs. 3.2 ± 2.0) were more affected, but without significance (p > 0.05). Pain scores were comparable (3.2 ± 2.1 vs. 3.5 ± 2.2, p = 0.12). Hormonotherapy was strongly associated with global sexual dysfunction compared with no hormonotherapy (OR 4.20, 95% CI 1.69-10.47, p = 0.01). Among users, 63.4% received tamoxifen and 36.6% aromatase inhibitors (Table 5).

**Table 5:** Sexual dysfunction according to hormonotherapy (n=200) SD: Standard Deviation; OR: Odds Ratio; CI: Confidence Interval; FSFI: Female Sexual Function Index OR (95% CI) derived from univariate logistic regression for global sexual dysfunction (FSFI ≤ 26.55). * p < 0.05 is considered statistically significant.

FSFI Domain	Hormonotherapy (n=110)	No Hormonotherapy (n=90)	p-value	OR (95 % CI)
Global dysfunction	94%	78%	0.01*	4.20 (1.69–10.47)
Desire (mean ± SD)	2.7 ± 1.9	3.3 ± 2.0	0.03*	—
Arousal (mean ± SD)	2.6 ± 1.9	3.2 ± 2.0	0.09	—
Orgasm dysfunction	56%	41%	0.07	—
Lubrication issues	51%	33%	0.04*	—
Pain (mean ± SD)	3.2 ± 2.1	3.5 ± 2.2	0.12	—

## Discussion

The breast represents not only an organ of femininity, motherhood, and sexuality but also a central component of women’s identity. Breast cancer remains the most common cause of cancer-related mortality among women worldwide, although advances in early detection and treatment have led to a steadily growing population of survivors [8]. In this context, we evaluated the sexual health of 200 sexually active women in remission from breast cancer. Our study revealed a strikingly high prevalence of sexual dysfunction, affecting 87% of participants, with sexual desire and arousal being the most impaired domains. These findings underscore that sexual health remains a neglected but crucial dimension of survivorship care.

The mean age of our participants was 50.3 years, with 74% between 41 and 67 years. This is consistent with previous African studies, where breast cancer is most commonly diagnosed around 50-60 years [9,10]. The predominance of sexual dysfunction in older women has been attributed to the combined impact of aging and treatment-induced menopause, which aggravates symptoms such as vaginal dryness and decreased libido [11].

Sociodemographic characteristics mirrored regional findings. Sixty percent of women lived in urban areas, and 40% had a university-level education, similar to data from Karoui et al. [12]. Partner characteristics also play a role: while 44% of partners in our cohort had a university degree, Shiferaw et al. reported much higher illiteracy rates among husbands in Ethiopia [13]. This variation illustrates how couple dynamics and education levels may affect sexual adjustment after cancer.

Reproductive history in our population showed a mean parity of 3.2 children. This aligns with findings from Falque-Pierrotin [14] but differs from the Tunisian series by Ahmed et al., where multiparity was more prevalent [3]. Prior studies have highlighted that reproductive history may shape both the psychological and physical experience of sexuality, either exacerbating fears of infertility and body changes or reinforcing intimacy through shared parenthood [15].

More than two-thirds of women were postmenopausal, nearly half due to treatment-induced ovarian failure. Premature menopause after oophorectomy or chemotherapy is well documented and strongly linked to sexual dysfunction [16]. Menopausal status, particularly treatment-induced menopause, likely acts as a partial confounder of these associations and merits stratified or multivariable analysis in future studies.

Treatment patterns in our cohort were in line with standard protocols: surgery in 83% of patients, chemotherapy in 92%, radiotherapy in 80%, and hormonotherapy in 55%. Breast reconstruction was rare (10%), considerably lower than the 28% reported by Vincent et al. [17]. Given the established role of reconstruction in improving body image and sexual recovery [18,19], these figures highlight an unmet need in Tunisian practice. These associations were clinically relevant, with odds ratios ranging from 2.86 (radical surgery) to 4.20 (hormonotherapy), highlighting the impact of treatment modalities on sexual function.

Our FSFI results (mean score 18.3 ± 11.6) align with those reported by Kufel-Grabowska et al. [20]. The 87% prevalence of dysfunction exceeds rates in some chronic disease populations, such as Tunisian women with chronic low back pain, where Fazaa et al. found 75% prevalence [21]. The highest burden was in desire (59%) and arousal (59.5%), consistent with studies linking these deficits to anxiety, fatigue, altered body image, and hormonal therapy [22,23]. Vaginal dryness and dyspareunia, reported by 43% and 56.5%, respectively, reflect the known impact of aromatase inhibitors and radiotherapy [22-24].

Beyond the overall prevalence, our comparative analysis highlighted the differential impact of treatment modalities. Radical surgery was associated with significantly poorer desire and arousal scores compared with conservative surgery, echoing reports that mutilating procedures have lasting consequences on sexual body image. Radiotherapy was linked to lubrication difficulties and pain, in line with studies documenting mucosal toxicity and fibrosis. Hormonotherapy users had the highest prevalence of dysfunction, particularly in desire and lubrication, reflecting the profound estrogen depletion induced by aromatase inhibitors and anti-estrogens. In contrast, chemotherapy showed only a trend toward lower scores, which did not reach significance, likely due to the small size of the comparator group. Together, these findings emphasize that not all treatments carry the same burden on sexual health, with hormonotherapy and radiotherapy exerting the most deleterious effects.

Despite these difficulties, more than two-thirds of women rated their relationships as satisfactory, highlighting the resilience of emotional bonds and the protective role of partner support [25,26].

Our findings reinforce the multidimensional nature of sexual health in breast cancer survivors. Beyond physical sequelae, issues of femininity, attractiveness, and intimacy weigh heavily on sexual function. Employment status, partner’s education, and reproductive history all emerged as important contextual factors. This underlines the need to adopt a bio-psycho-social framework when addressing sexuality in oncological care.

Although older and postmenopausal women tended to have higher rates of dysfunction, this association lost statistical significance after adjusting for treatment-induced menopause. Baseline sexual function prior to cancer diagnosis was not assessed, which limits the ability to distinguish pre-existing difficulties from treatment-related effects. These aspects highlight the importance of stratifying future analyses by menopausal status and considering pre-diagnosis sexual history in future research.

Clinical implications

Our findings highlight the need to integrate sexual health into routine breast cancer follow-up. Systematic use of the FSFI can help identify dysfunction early, while training oncologists and gynecologists in sexual history-taking and referral pathways may improve quality of life and relationship satisfaction.

Study highlights, strengths, and limitations

This study provides one of the largest multicenter datasets from Tunisia examining sexual health in breast cancer survivors, using the validated Arabic version of the FSFI. By integrating sociodemographic, clinical, and treatment-related data, it offers a comprehensive overview of the multidimensional impact of breast cancer on sexuality. Key findings include the very high prevalence of sexual dysfunction (87%), the predominance of desire and arousal disorders, and the strikingly low rate of breast reconstruction, which together highlight major gaps in survivorship care.

Nevertheless, several limitations must be acknowledged. The cross-sectional design precludes causal inference, and the use of convenience sampling may have introduced selection bias. The use of a convenience sampling strategy may limit generalizability. Nevertheless, consecutive enrollment from three centers helped reduce selection bias.

Almost half of the participants had low literacy, which may have affected self-report accuracy despite assistance provided. In addition, the absence of partner perspectives limited assessment of relational dynamics, which are essential to understanding sexuality. Moreover, the study did not explore whether participants discussed their sexual concerns with healthcare providers or whether any treatment or counseling was offered, which limits insight into the management of sexual dysfunction in this population.

Most participants belonged to middle or lower socioeconomic strata, reflecting the public hospital setting of recruitment. Limited financial resources and restricted access to psychosexual counseling may exacerbate the effects of treatment-related dysfunction. Furthermore, socioeconomic constraints often interact with cultural norms to hinder open discussion of sexual issues within couples.

Cultural factors specific to Tunisian society, including stigma around sexuality and gender roles, may influence self-reporting and satisfaction. Culturally sensitive counseling and partner involvement could enhance management.

Future studies should adopt longitudinal and interventional designs to better capture trajectories of sexual recovery and evaluate rehabilitation programs. Including partners’ perspectives and integrating qualitative research could deepen understanding of sexual adaptation and relational dynamics after breast cancer. Exploring whether women discuss sexual concerns with healthcare providers and assessing the knowledge and attitudes of oncology and gynecology professionals regarding sexual dysfunction would also provide valuable insight for developing effective, culturally sensitive interventions.

Clinically, our findings emphasize the urgent need to integrate sexual health assessment and counseling into oncology follow-up, ensure systematic information on reconstructive options, and develop culturally adapted interventions to improve quality of life in breast cancer survivors.

## Conclusions

Sexual dysfunction is frequent among Tunisian breast cancer survivors and is influenced by both treatment-related and sociocultural factors. The high prevalence observed underscores the need for comprehensive survivorship care including systematic sexual health assessment. Multidisciplinary approaches and culturally adapted counseling should be encouraged to improve women’s well-being and marital satisfaction after cancer.

Beyond its high frequency, this dysfunction reflects the persistent under-recognition of sexual health in oncologic follow-up, particularly in low- and middle-income settings. Routine screening using standardized tools such as the FSFI could facilitate early identification and timely management. Training programs for oncologists and gynecologists in sexual communication are essential to overcome stigma and foster patient-centered care. Further longitudinal and interventional studies are warranted to explore causal pathways, evaluate treatment-specific effects, and develop culturally sensitive rehabilitation strategies tailored to Tunisian and North African women.
